# Cost–utility analysis of using high-intensity statin among post-hospitalized acute coronary syndrome patients

**DOI:** 10.1186/s43044-024-00478-2

**Published:** 2024-04-14

**Authors:** Pramitha Esha Nirmala Dewi, Montarat Thavorncharoensap, Bangunawati Rahajeng

**Affiliations:** 1 Doctor of Philosophy Program in Social, Economic, and Administrative Pharmacy, Department of Pharmacy, Faculty of Pharmacy, Mahidol University, Bangkok, Thailand; 2 Department of Pharmacy Profession, Faculty of Medicine and Health Sciences, Universitas Muhammadiyah Yogyakarta, Bantul, Indonesia; 3 Social and Administrative Pharmacy Excellence Research (SAPER) Unit, Department of Pharmacy, Faculty of Pharmacy, Mahidol University, Bangkok, Thailand; 4 Health Technology Assessment Graduate Program, Mahidol University, Bangkok, Thailand

**Keywords:** Acute coronary syndrome, Cost–utility analysis, High-intensity stain, Cost per quality-adjusted life year

## Abstract

**Background:**

Post-hospitalized acute coronary syndrome (ACS) patients in Indonesia National Insurance does not pay for the use of high-intensity statin (HIS) for secondary prevention after ACS hospitalization. Moreover, a cost–utility analysis needs to be conducted to evaluate the cost-effectiveness of prescribing HIS and low-to-moderate-intensity statin (LMIS) per quality-adjusted life year (QALY). This study aimed to estimate the cost–utility of long-term HIS treatment in post-hospitalized ACS patients in Indonesia compared to current practice.

**Results:**

This study compared the economic outcomes of long-term HIS and LMIS in Indonesian post-hospitalized ACS patients. A lifetime Markov model predicted ACS-related events, costs, and QALY from a payer perspective. A systematic review estimated treatment-specific event probabilities, post-event survival, health-related quality of life, and Indonesia medical-care expenses from published sources. This study conducted probabilistic sensitivity analysis (PSA) using 1000 independent Monte Carlo simulations and a series of one-way deterministic sensitivity analyses utilizing a tornado diagram. The economic evaluation model proved that intensive HIS treatment can increase per-patient QALYs and care expenditures compared to LMIS. The use of HIS among post-hospitalized ACS patients had ICER 31.843.492 IDR per QALY gained, below the Indonesia willingness-to-pay (WTP) for terminal disease and life-saving treatment.

**Conclusion:**

From the Indonesia payer perspective, using HIS for post-hospitalized ACS patients in Indonesia is cost-effective at 31.843.492 IDR per QALY gained.

## Background

Cardiovascular disease is the highest rate of total burden noncommunicable disease worldwide during these five recent years as the leading cause of disability-adjusted life year (DALY) loss globally [1]. Cardiovascular diseases account for an estimated one-third of global mortality, with ischemic heart disease (IHD) being the specific cause of 7.5 million of these fatalities [2]. Acute coronary syndromes (ACSs) and sudden death account for the majority of IHD-related fatalities annually, amounting to 1.8 million. ACS, in general, increases incidence with age; however, this occurs 7–10 years earlier in men on average than in women. According to the American Heart Association, one heart attack occurs approximately every 41 s [3]. Age-standardized mortality rates (ASMRs) for ACS were highest in lower-income global regions in 2020 for both sexes [4]. Some recent guidelines recommend the use of statins as the major therapy for atherosclerotic cardiovascular disease (ASCVD), including ACS [5]. The use of statins as an important medication in the primary and secondary prevention of vascular diseases has been applied to patients with ACS [6]. Ensuring consistent adherence to statin therapy decreases the likelihood of experiencing an initial cardiovascular disease (CVD) event and subsequent CVD events in high-risk individuals (primary prevention and secondary prevention, respectively) [7]. The effort to reduce low-density lipoprotein-cholesterol (LDL-C) levels is closely related to diminishing the risk of recurrence of cardiovascular events among ACS patients. The prescriber should observe the clinical outcomes data, the cost aspect, and the patient’s quality of life during the ACS therapy.

Financial and humanistic aspects are becoming pivotal concerns for chronic diseases and clinical outcomes. These concerns are due to the patients experiencing the therapy for a long period, so the prescriber should be aware of how to optimize the statin therapy during the treatment period. Statin should be prescribed to high-risk cardiovascular event patients. Based on their ability to reduce LDL-C levels, statins are classified as high-intensity, moderate-intensity, or low-intensity. LDL-C levels are reduced by at least 50% with high-intensity statins, such as atorvastatin (40–80 mg daily) and rosuvastatin (20–40 mg daily). Moderate-intensity statins, including atorvastatin (10–20 mg daily), rosuvastatin (5–10 mg daily), simvastatin (20–40 mg daily), and others, decrease LDL-C levels by 30–50%. Low-intensity statins, including Simvastatin (10 mg daily), Pravastatin (10–20 mg daily), and others, decrease LDL-C levels by less than 30%. The selection of statin dosage is contingent upon the patient's cardiovascular health and risk factors. Low-intensity statins may be more suitable for patients with lesser risk factors or those who cannot tolerate higher doses, whereas high-intensity statins are generally advised for individuals who are at a heightened risk for cardiovascular events [8, 9].

Some studies revealed that the use of high-intensity statin (HIS) is still underused for those populations and for secondary prevention in some countries [10, 11]. Based on Indonesia’s national list of essential medicines and Indonesian Case Base Groups (INA-CBGs), the use of HIS will only be covered by the government in the hospitalized setting and three weeks after discharge [12]. Moreover, it needs to conduct the cost–utility analysis to evaluate the cost-effectiveness of prescribing HIS compared to low-to-moderate-intensity statin (LMIS) per quality-adjusted life year (QALY) gained among the post-hospitalized patients for a lifetime period in the setting of Indonesia’s national health insurance.

This study conducts cost simulation to present the cost-effectiveness of using HIS as secondary prevention among patients with post-hospitalized ACS compared to LMIS. Markov model is used for the current study by applying lifetime horizon and payer perspective to evaluate. Cost-effectiveness study from a payer standpoint pertains to the expenditures accrued by a particular payer, such as a government agency, insurance company, or healthcare provider [13]. This study focused on providing data about the benefit of covering the need for lifetime consumption of HIS as a secondary prevention among post-hospitalized ACS patients, which is not covered by Indonesian government insurance (BPJS). It only covered the use of HIS in the hospitalized setting until three weeks after discharge. The lifetime use of HIS among post-hospitalized ACS patients is recommended by the updated guideline [12]. When evaluating the cost-effectiveness of interventions, payer perspective is frequently applied in cost-effectiveness studies. The payer is typically concerned with the impact of a treatment or intervention on patient health outcomes and the costs associated with it [14, 15]. Therefore, this study aimed to estimate the cost–utility of long-term use of high-intensity statin among post-hospitalized patients with acute coronary syndrome compared with current practice in Indonesia.

## Methods

### Research design

**Fig. 1:**
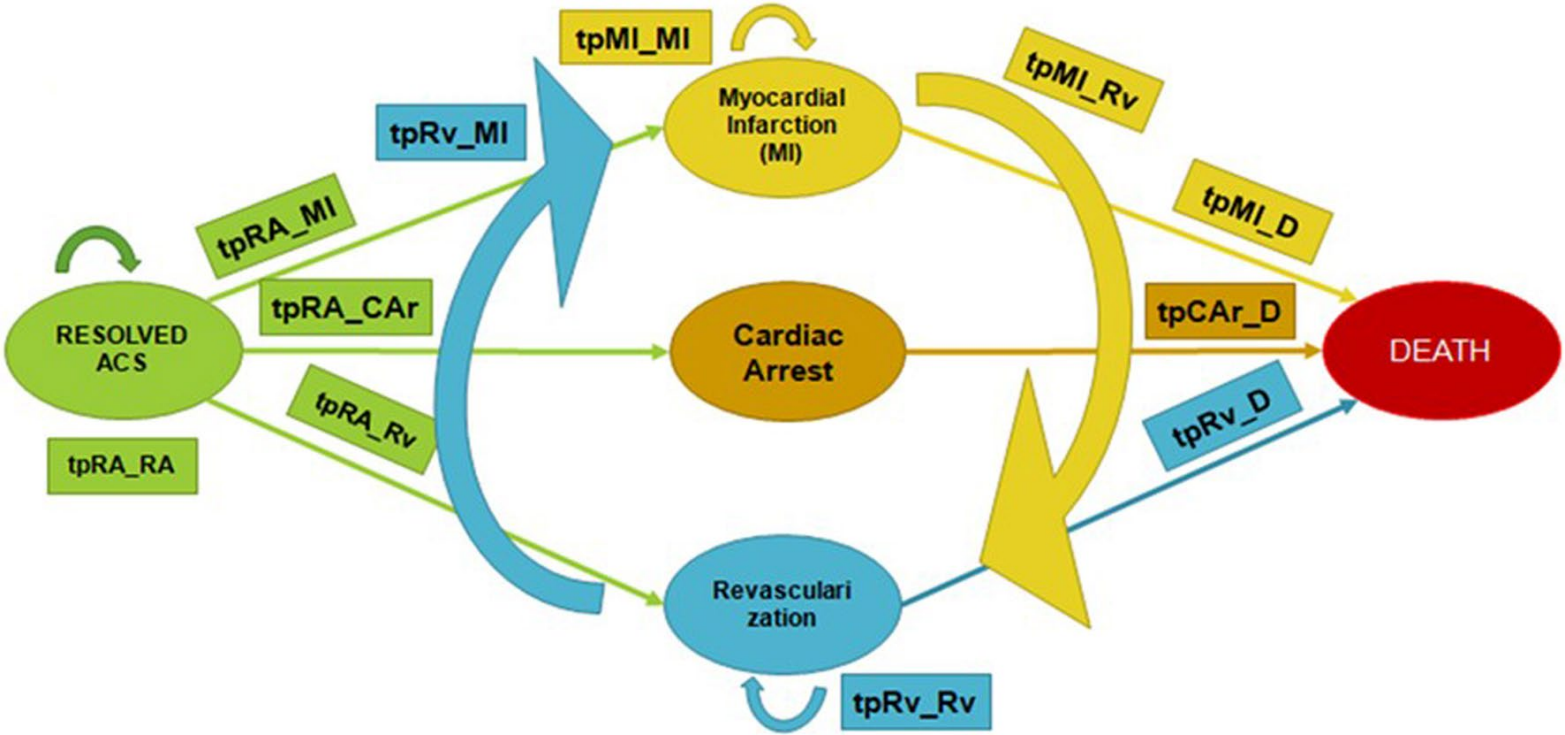
Markov model states and possible transitions

The applied economic assessment in this study was a cost–utility analysis in which the incremental cost per QALY gained was utilized to determine cost-effectiveness. This study employed techniques of the Markov model to construct a state-transition model of the outcomes and costs of secondary cardiovascular prevention. Based on the type of statin therapy received (HIS/LMIS), the model predicts the likelihood of experiencing Resolved ACS (RA) and major cardiovascular events myocardial infarction (MI), revascularization (RV), cardiac arrest (CAr)], and death as seen in Fig. 1. All analyses in this study were performed from the payer perspective, which refers to Indonesia's national health insurance.

### Parameters

In regard to obtaining relevant input parameters and studies that could support the current study method, a systematic review was conducted. PubMed and Scopus databases were searched from conception to February 2022. The search terms and strategies were using keywords as follows: (post hospitalized) and (acute coronary syndrome) and (high-intensity statin or Low-Intensity Statin or Moderate Intensity Statin or Simvastatin or Atorvastatin or Rosuvastatin) and (Major Adverse Cardiac Events or Cardiac Event Survival) and (Cost Benefit Analysis or Cost-Effectiveness Analysis or Cost-Utility Analysis). Any kinds of studies published in English were selected if they met the inclusion criteria: (i) any kind of cost analysis, (ii) used the post-hospitalized ACS patient as the sample/participant, (iii) had at least one major adverse cardiac event (MACE) as the end point of statin intervention. Studies were excluded if there was insufficient information related to statin's name and dose. Each study's performance was checked using Cheers Checklist [16].

### Transition probability

The Markov model was composed of several distinct health states defined according to major CVD event status (Resolved ACS, Cardiac Arrest, Myocardial Infarction, Revascularization, and Death) (Fig. 1). The parameter description is indicated in Table 1. Within a Markov model, the health states represent various illness stages, treatment choices, or end results. The transition probabilities quantify the probability of transitioning from one health state to another during a certain period of time. Utilities, or quality of life (QoL) metrics, are allocated to each health state to quantify the effect of the health state on the patient's well-being [17, 18]. Measures of utility (a summary measure of quality of life on a zero-to-one scale) and economic cost are assigned to each health state. Patients in major event states are subjected to the long-term utility and mortality consequences of their specific cardiovascular event(s) [19].

**Table 1:** Event probabilities, cost, hazard ratio, and utility used in the model

Name	Value	Parameter description	SE	Ref
drC	0.03	Discounting rate for costs		WHO 2015
drO	0.03	Discounting rate for outcomes		WHO 2015
*Transitional probabilities*				
tpRA_RA	0.952	Transition probability from Resolved ACS to Stay Resolved ACS	0.095	
tpRA_MI	0.012	Transition probability from RA to MI	0.001	Taylor et al. [19]
tpRA_CAr	0.001	Transition probability from RA to Cardiac Arrest	0.001	Taylor et al. [19]
tpRA_Rv	0.035	Transition probability from RA to Revascularization	0.001	Taylor et al. [19]
tpMI_MI	0.049	Transition probability from MI to MI	0.010	Taylor et al. [19]
tpMI_Rv	0.027	Transition probability from MI to Revascularization	0.004	Taylor et al. [19]
tpMI_D_30to34	0.007	Transition probability from MI to Death age 30–34	0.001	Taylor et al. [19]
tpMI_D_35to39	0.009	Transition probability from MI to Death age 35–39	0.001	Taylor et al. [19]
tpMI_D_40to44	0.014	Transition probability from MI to Death age 40–44	0.001	Taylor et al. [19]
tpMI_D_45to49	0.020	Transition probability from MI to Death age 45–49	0.002	Taylor et al. [19]
tpMI_D_50to54	0.032	Transition probability from MI to Death age 50–54	0.003	Taylor et al. [19]
tpMI_D_55to59	0.050	Transition probability from MI to Death age 55–59	0.005	Taylor et al. [19]
tpMI_D_60to64	0.084	Transition probability from MI to Death age 60–64	0.008	Taylor et al. [19]
tpMI_D_65to69	0.131	Transition probability from MI to Death age 65–69	0.013	Taylor et al. [19]
tpMI_D_70to74	0.205	Transition probability from MI to Death age 70–74	0.021	Taylor et al. [19]
tpMI_D_75to79	0.326	Transition probability from MI to Death age 75–79	0.033	Taylor et al. [19]
tpMI_D_80to84	0.508	Transition probability from MI to Death age 80–84	0.051	Taylor et al. [19]
tpCAr_D_30to34	0.004	Transition probability from Cardiac Arrest to Death age 30–34	0.000	Taylor et al. [19]
tpCAr_D_35to39	0.005	Transition probability from Cardiac to Death age 35–39	0.001	Taylor et al. [19]
tpCAr_D_40to44	0.008	Transition probability from Cardiac Arrest to Death age 40–44	0.001	Taylor et al. [19]
tpCAr_D_45to49	0.012	Transition probability from Cardiac Arrest to Death age 45–49	0.001	Taylor et al. [19]
tpCAr_D_50to54	0.018	Transition probability from Cardiac Arrest to Death age 50–54	0.002	Taylor et al. [19]
tpCAr_D_55to59	0.028	Transition probability from Cardiac Arrest to Death age 55–59	0.003	Taylor et al. [19]
tpCAr_D_60to64	0.048	Transition probability from Cardiac Arrest to Death age 60–64	0.005	Taylor et al. [19]
tpCAr_D_65to69	0.074	Transition probability from Cardiac Arrest to Death age 65–69	0.007	Taylor et al. [19]
tpCAr_D_70to74	0.116	Transition probability from Cardiac Arrest to Death age 70–74	0.012	Taylor et al. [19]
tpCAr_D_75to79	0.185	Transition probability from Cardiac Arrest to Death age 75–79	0.019	Taylor et al. [19]
tpCAr_D_80to84	0.288	Transition probability from Cardiac Arrest to Death age 80–84	0.029	Taylor et al. [19]
tpRv_Rv	0.135	Transition probability from Revascularization to Revascularization	0.009	Taylor et al. [19]
tpRv_MI	0.396	Transition probability from Revascularization to MI	0.024	Taylor et al. [19]
tpRv_D_30to34	0.004	Transition probability from Revascularization to Death age 30–34	0.000	Taylor et al. [19]
tpRv_D_35to39	0.005	Transition probability from Revascularization to Death age 35–39	0.001	Taylor et al. [19]
tpRv_D_40to44	0.007	Transition probability from Revascularization to Death age 40–44	0.001	Taylor et al. [19]
tpRv_D_45to49	0.011	Transition probability from Revascularization to Death age 45–49	0.001	Taylor et al. [19]
tpRv_D_50to54	0.017	Transition probability from Revascularization to Death age 50–54	0.002	Taylor et al. [19]
tpRv_D_55to59	0.027	Transition probability from Revascularization to Death age 55–59	0.003	Taylor et al. [19]
tpRv_D_60to64	0.045	Transition probability from Revascularization to Death age 60–64	0.005	Taylor et al. [19]
tpRv_D_65to69	0.071	Transition probability from Revascularization to Death age 65–69	0.007	Taylor et al. [19]
tpRv_D_70to74	0.111	Transition probability from Revascularization to Death age 70–74	0.011	Taylor et al. [19]
tpRv_D_75to79	0.176	Transition probability from Revascularization to Death age 75–79	0.018	Taylor et al. [19]
tpRv_D_80to84	0.274	Transition probability from Revascularization to Death age 80–84	0.027	Taylor et al. [19]
*Cost parameters*				
*Direct Costs per Health State*				
dmcRA	Rp 19,728,100	Direct medical costs associated with Resolved ACS	Rp 197,281	INA-CBGs 2016
dmcMI	Rp 12,118,800	Direct medical costs associated with Myocardial Infarction	Rp 121,188	INA-CBGs 2016
dmcCAr	Rp 7,041,400	Direct medical costs associated with Cardiac Arrest	Rp 70,414	INA-CBGs 2016
dmcRv	Rp 40,024,100	Direct medical costs associated with Revascularization	Rp 400,241	INA-CBGs 2016
*Cost of Interventions*				
cHIS	Rp 3,908,568.00	Direct Medical Cost of using High-Intensity Statin per year	Rp 390,856.80	Indonesia Secondary Hospital Type B
cNHIS	Rp 1,474,656.00	Direct Medical Cost of using Moderate Statin per year	Rp 147,465.60	Indonesia Secondary Hospital Type B
*High-Intensity Statin Efficacy*				
HR_HIS_MI	0.77	HR for MI events by using HIS	0.07	Taylor et al. [19]
HR_HIS_CAr	1.07	HR for Cardiac Arrest events by using HIS	0.11	Taylor et al. [19]
HR_HIS_Rv	0.73	HR for Revascularization events by using HIS	0.04	Taylor et al. [19]
*Utility parameters*				
uRA	0.78	Quality of life for Resolved ACS	0.078	Lin et al. [20]
uMI	0.65	Quality of life for Myocardial Infarction	0.065	Lin et al. [20]
uCAr	0.68	Quality of life for Cardiac Arrest	0.068	Lin et al. [20]
uRv	0.78	Quality of life for Revascularization	0.078	Taylor et al. [19]

Values indicate a numerical reference for each parameter that were derived from previous study to be apply in the current study model

SE (standard error) signifies the lack of certainty in the predicted effect size from each chosen reference

Estimation of the model involves predicting and tracking patients’ transitions across these health states in 1-year intervals, in a Markov process, and tallying their CVD events, life-years, QALYs, and costs over the course of their lifetimes [20]. The model was calculating the transitional probabilities started from age 30 years old.

### Utility and cost

Indonesian data were used to estimate the cost-effectiveness. Model parameters included drug costs, event costs, hazard ratio, and population mortality. The target population was a hypothetical cohort of 1000 Indonesian patients with post-hospitalized ACS. Model parameters that were kept fixed across all countries included cardiovascular event rates, treatment efficacy, utility weights, and the effects of cardiovascular events on survival. The Markov model will be adapted to simulate a hypothetic cohort of Post-Hospitalized ACS patients who received low to moderate-intensity statin compared to those who received high-intensity statin. Each cost intervention was collected from one of the Indonesia Secondary Hospital data per visit per patient in 2022. All costs were considered in Indonesian Rupiah and then converted to USD only for the cost-effectiveness ratio (ICER)/QALY based on the exchange rate by March 31, 2022. All values used in the model for the current study, including probabilities, cost, hazard ratio, and utility, are displayed in Table 1. A lifetime period was used for the simulation in this study. The annual discount rate for expenses and utilities in the base cases was established at 3% by the methodological norms for pharmacoeconomic evaluations. The primary cost-effectiveness outcome was incremental cost per QALY gained for patients receiving high-intensity statin compared to LMIS per quality-adjusted life year (QALY) gained among the post-hospitalized patients for a lifetime period in the setting of Indonesia’s national health insurance.

Direct Medical Cost per Health State was obtained from Indonesia-Case Based Groups 2016 (INA-CBGs) for Government Hospital Type B. All Direct Medical Cost per Health States are referred to moderate state and using 2nd Class Room facilities. For this model, all patients started from the stable ACS and then move to other health states as the second events.

### Sensitivity analysis

In order to incorporate uncertainties and model assumptions, sensitivity analysis was conducted for each variable across its estimated range. In order to accomplish this, we simulated the daily administration of high-intensity statin (HIS) and low- to moderate-intensity statin (LMIS) to hospitalized patients. HIS is defined as administering atorvastatin 40 mg or rosuvastatin 20 mg. LMIS is defined as the use of any dose of simvastatin, including lower doses of atorvastatin/rosuvastatin (the annual cost was 1.474.656 IDR and 3.908.568 IDR for LMIS and HIS, respectively, during 2022). To more precisely assess the accuracy of our cost-effectiveness estimates, this study conducted probabilistic sensitivity analysis (PSA) by using 1000 independent Monte Carlo simulations and a series of one-way deterministic sensitivity analyses utilizing a tornado diagram. In each simulation, a random sample was taken from each variable across their respective range of estimates.

## Results

A total of 224 articles were identified from PubMed and Scopus. A hundred and eleven duplicates were removed, leaving 113 articles for the title and abstract screening. Then, 60 unrelated articles were excluded, resulting in 53 articles for full-text screening. Finally, 8 articles were eligible to include in the review. The data related to the selected studies are presented in Table 2.

**Table 2:** Selected references

References	Intervention	Comparator	Perspective	Cost	Outcome	Model	Time Horizon	Discount
Barrios et al. [21]	Polypill	Multiple monotherapy (Atorvastatin)	Payer	Direct	MACE	Markov	10 years	3%
Almalki et al. [22]	Simvastatin 40 mg + Ezetimibe 10 mg	Simvastatin 40 mg	Payer	Direct	MACE	Markov	5 years 10 years Lifetime	3%
Gómez-Gerique et al. [23]	Atorvastatin 80 mg	Placebo	Payer	Direct	MACE	Markov	Lifetime	3.5%
Ademi et al. [24]	100% statin coverage (Low-Intensity Statin)	82% statin coverage (Low-Intensity Statin)	Government	Direct	MACE	Markov	5 years	5%
Lazar et al. [25]	Low-intensity statin (low cost)	Suggested statin by ATP III	Societal	Direct	MACE	Markov	30 years	3%
Chan et al. [26]	High Dose Statin	Conventional Statin	Societal	Direct	MACE	Markov	Lifetime	3%
Taylor et al. [19]	Atorvastatin 80 mg	Atorvastatin 10 mg	Payer	Direct	MACE	Markov	Lifetime	3.5%
Lin et al. [20]	Moderate-Intensity Statin	No Statin	Payer	Direct	MACE	Markov	10 ears	3%

The time horizon refers to the duration during which costs and impacts are assessed

Discount is a method frequently employed in cost-effectiveness analysis for "fairly" comparing programs whose outcomes and costs transpire at dissimilar points in time

Based on the result above, most of the study used the payer perspective to analyze the cost-effectiveness of using statin. Most of the studies used life time horizon to run the Markov Model. Thus, Markov model for current study was applying life time horizon and payer perspective to evaluate cost-effectiveness of using HIS as secondary prevention for post-hospitalized ACS when compared with LMIS.

### Base case analysis

The results are presented in terms of incremental cost-effectiveness ratio (ICER), which was the difference in cost divided by the difference in QALY between HIS and LMIS treatment. Additionally, the simulated results of 1000 patients over lifetime years were reported. Based on the result on the deterministic and probabilistic analysis results, high-intensity statin therapy accrued more drug costs annually (27.375.930–34.094.257 IDR) compared to low-to-moderate-intensity statin therapy.

**Fig. 2:**
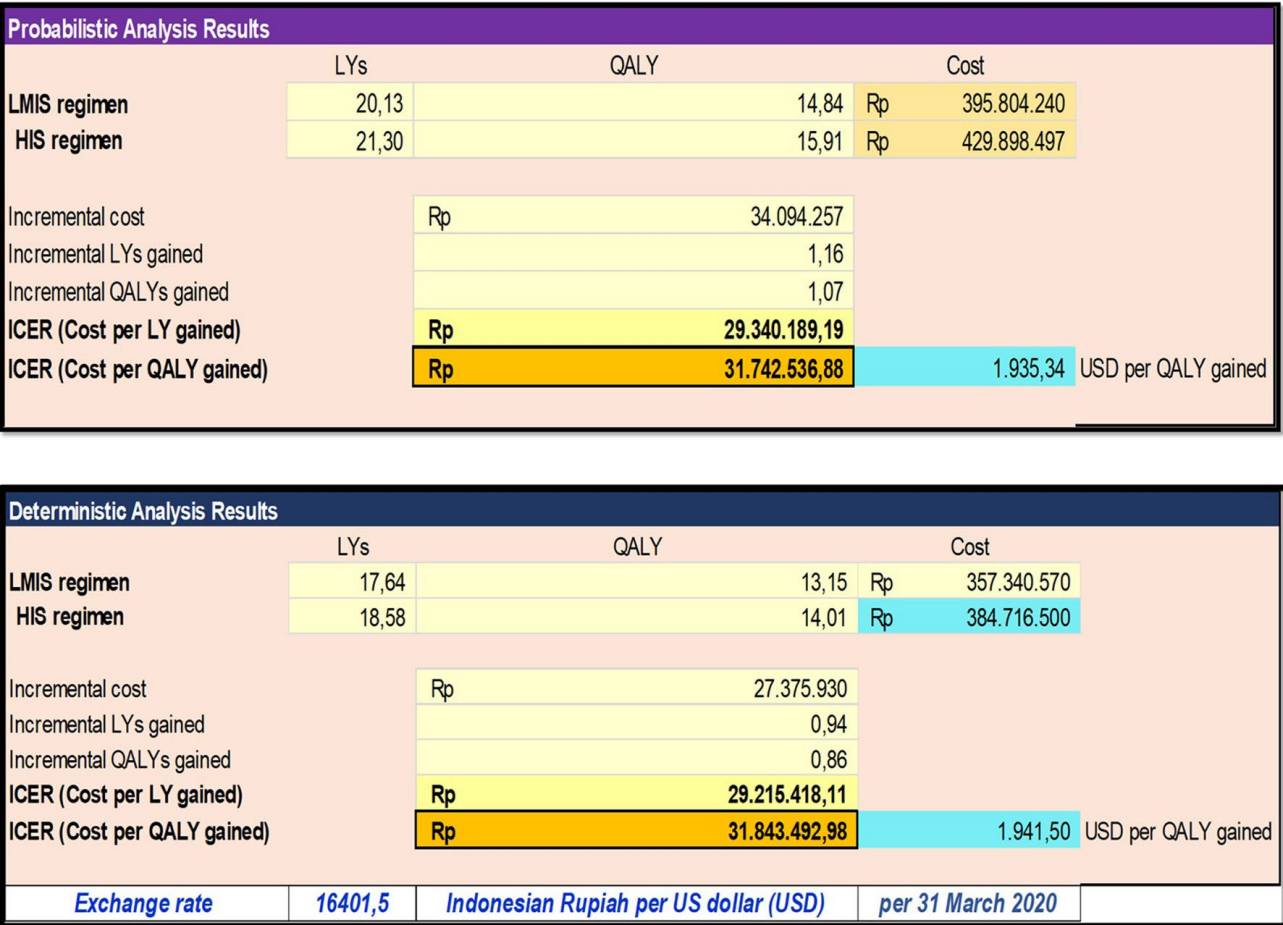
Deterministic and probabilistic analysis results

The incremental cost-effectiveness ratio (ICER) was 31.843.492,98 IDR and 31.742.536,88 IDR based on deterministic and probabilistic analysis, respectively, shown in Fig. 2. Refer to the Indonesia gross domestic product (GDP) (4193.109 USD in Dec 2019), the ICER of HIS intervention treatment is lower than one Indonesia GDP, so it can be considered highly cost-effective if it is applied as secondary prevention among post-hospitalized ACS patients for lifetime consumption from payer perspective.

### Sensitivity analysis

**Fig. 3:**
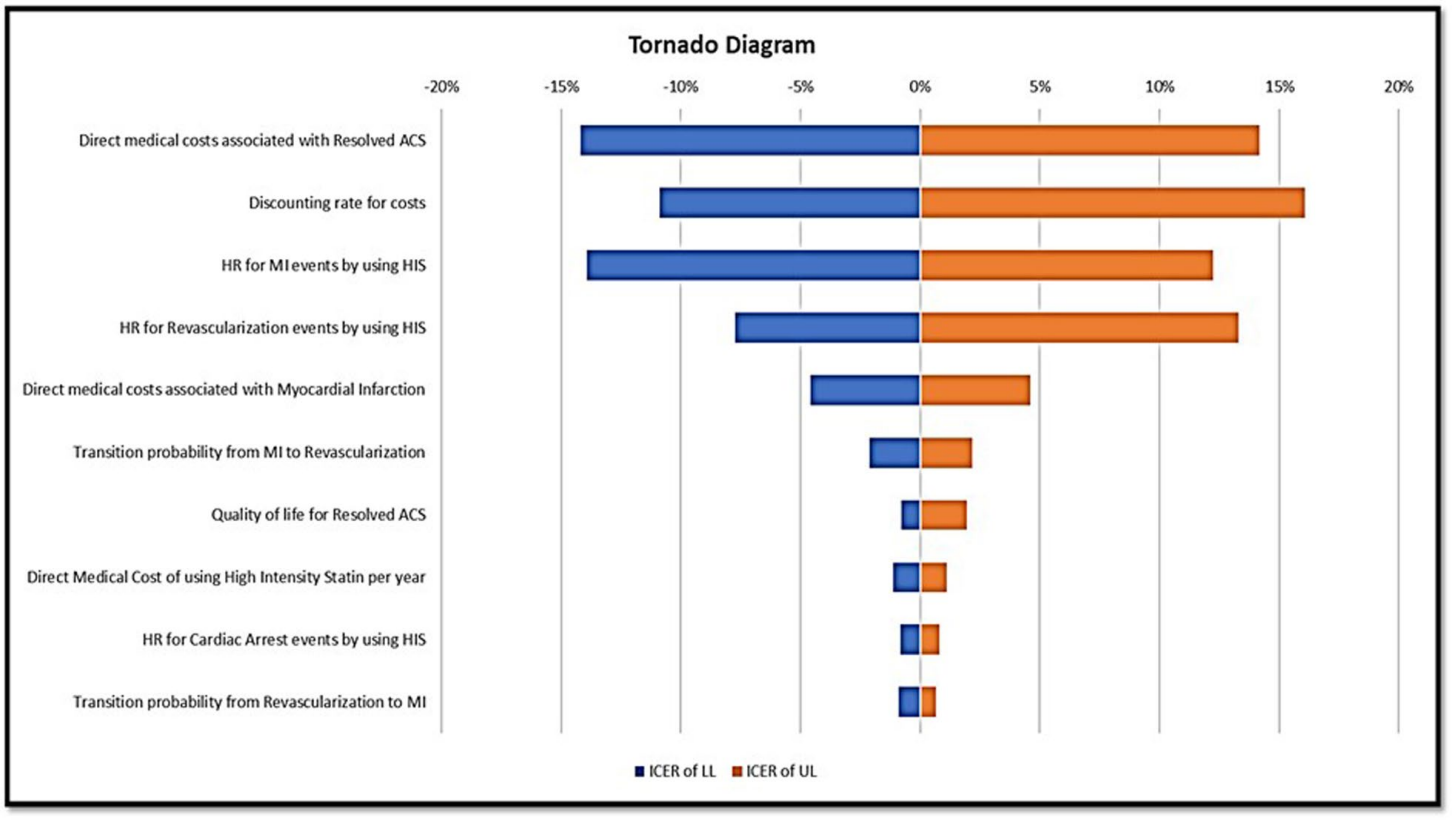
Tornado diagram presenting the one-way sensitivity analysis results

This study presents a series of one-way deterministic sensitivity analyses using a tornado diagram, as shown in Fig. 3. The ICER significantly increased with the elevated direct medical costs associated with resolved ACS. The ICER was sensitive to other parameters, such as Discounting Rate for Cost and Hazard Ratio for Myocardial Infarction events following Revascularization events among post-hospitalized ACS patients who received HIS. These analyses indicate that the model results are most sensitive to the direct medical costs associated with resolved ACS. The results are least sensitive to the utility values among patients with resolved ACS, the direct medical cost of using HIS, which is spent annually, and the Hazard Ratio of Cardiac Arrest among post-hospitalized ACS patients who received long-term HIS.

**Fig. 4:**
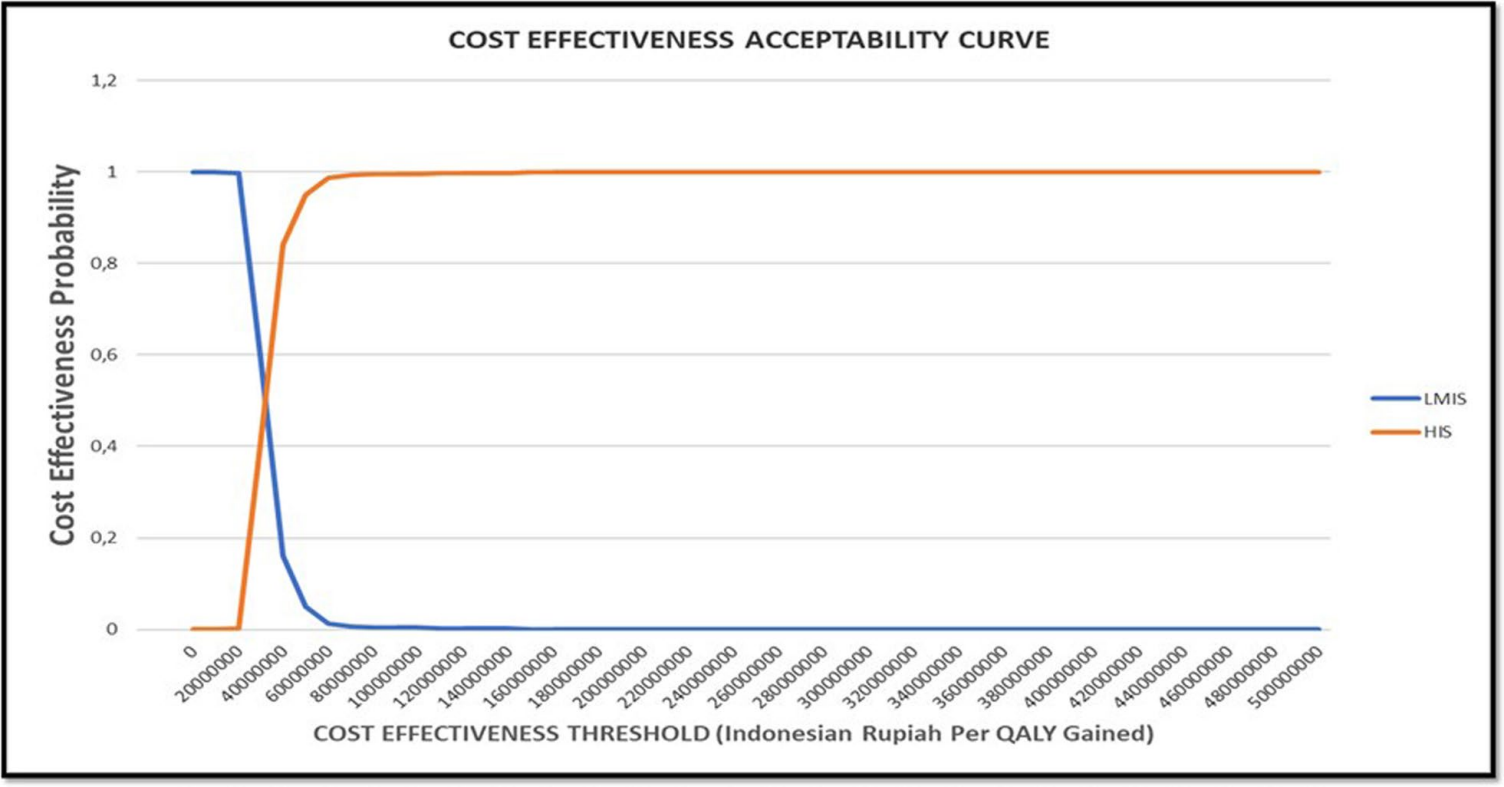
Probability that the use of HIS is cost-effective when compared to LMIS based on probabilistic sensitive analysis

The results of the probabilistic model analyses for Indonesia are displayed in the cost-effectiveness acceptability curves in Fig. 4. These curves indicate that the lifetime use of HIS among the post-hospitalized ACS patients is cost-effective in 0.99 (99%) of simulations at both thresholds of three times Indonesia GDP (206.319.831,79 IDR) per capita per QALY and WTP of life-saving disease in Indonesia (192.514.839 IDR) per QALY. Therefore, based on the Cost-Effectiveness Acceptability Curve, this study can assume that at both referred willingness to pay threshold per event avoided, there is a 99% probability that high-intensity statin long-term treatment among post-hospitalized ACS patients would be cost-effective.

**Fig. 5:**
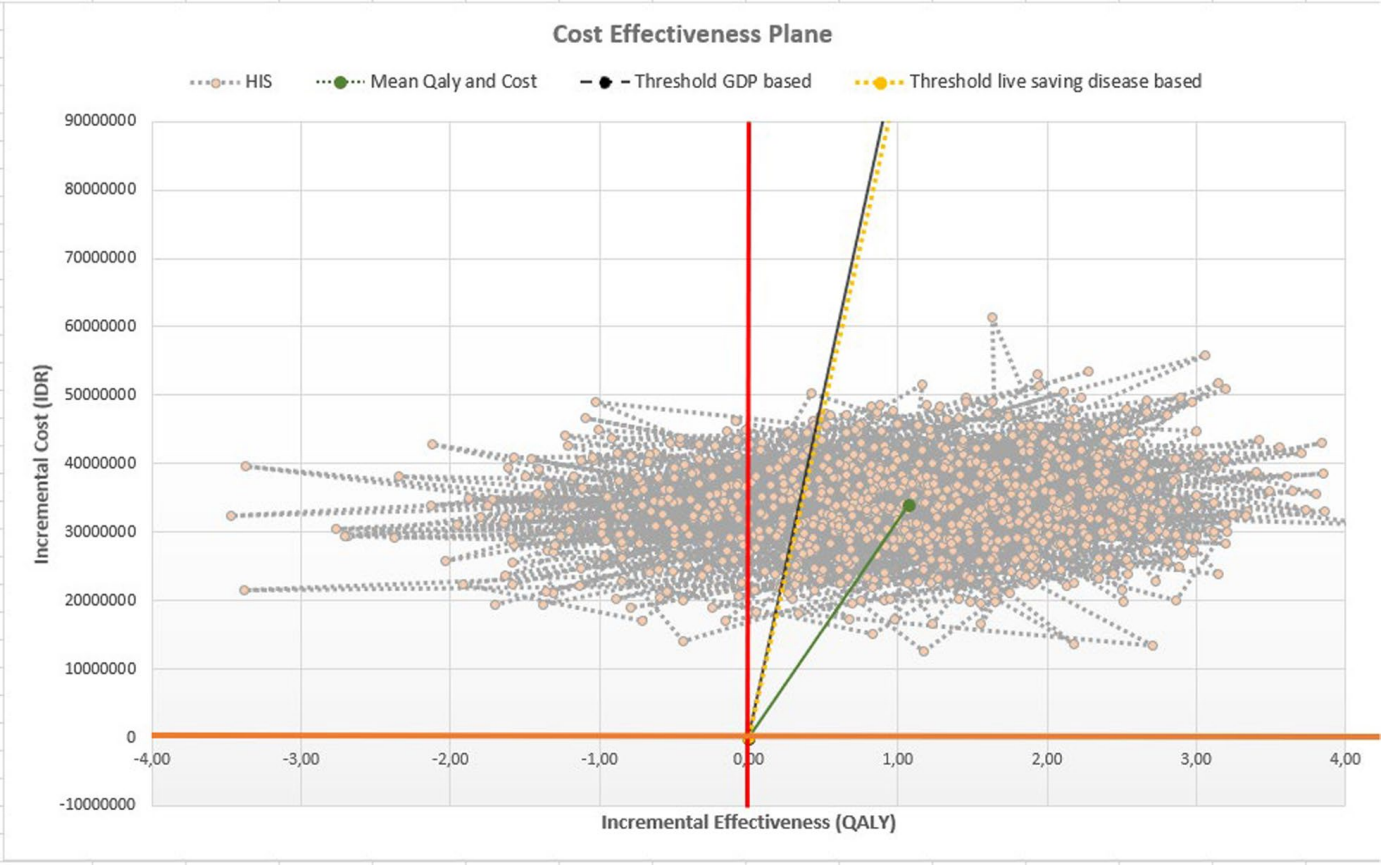
Plot of 1000 bootstrap samples showing the incremental cost between HIS and LMIS on *Y*-axis versus the incremental effectiveness on *X*-axis

A scatterplot of 1000 repetitions of bootstrap illustrates the uncertainty surrounding the estimation of the cost-effectiveness ratio (Fig. 5). All effectiveness points lie to the right of the vertical axis, indicating relative certainty regarding incremental effectiveness using HIS treatment over a lifetime. The points cross the horizontal axis, reflecting uncertainty about whether HIS treatment is dominant or improves the effectiveness point at additional cost. This study can report the probabilistic sensitivity analysis results through the cost-effectiveness plane. The four-quadrant diagram illustrates the incremental cost and effect (QALYs) of high-intensity statin treatment compared to low-to-moderate-intensity statin in the 1000 Monte Carlo simulations. The black diagonal line partitioning the plane represents the ceiling ratio for decision-making and defines the cost-effectiveness acceptability region based on Indonesia's GDP, and the yellow line based on Indonesia's willingness to pay for life-saving disease. The points below the diagonal line were cost-effective at a willingness-to-pay (WTP) threshold of Indonesia's GDP (206.319.831,79 IDR) per capita per QALY and WTP of life-saving disease in Indonesia (192.514.839 IDR) per QALY.

## Discussion

The systematic review process was utilized in this economic evaluation study, which utilized data from prior studies with the required values for the current investigation. After that, the Markov model was constructed using the data acquired from the carefully chosen articles. Unfortunately, only a few studies could provide the required data. Thus, a future study in Southeast Asia, specifically in Indonesia, should be conducted to provide such data for developing the same model. A comparison was made between the economic benefits of prescribing HIS for post-hospitalized patients with ACS and LMIS. The model was designed to illustrate this comparison. Patients diagnosed with acute coronary syndrome are given statins for both primary and secondary prevention of major adverse cardiac events [27]. A higher cost of HIS compared to LMIS was found in this study. However, the World Health Organization's (WHO's) Choosing interventions that are cost-effective (CHOICE) project defined interventions for which the cost per QALY gained is less than the gross domestic product (GDP) per capita is highly cost-effective and between one and three times GDP per capita as cost-effective [28]. In our scenario analysis, given that all statin is used once a day for life by the patient since the ACS hospitalization, based on the result, this study reports ICER per QALY below the Indonesia WTP for a terminal disease, moderate and live-saving treatment as mentioned in the study conducted by Kristina et al. (2018) [29] in Indonesia. By also referring to the previous study conducted by Tri Murti Andayani [30], this study reported the WTP in Indonesia for live-saving disease and terminal illness were 192,514,839 IDR (SD = 301,386,928); 194,976,141 IDR (SD = 350,974,703), respectively, which still much higher than ICER of HIS intervention by the model in this study. Therefore, the use of high-intensity statin is supposed to be applicable to post-hospitalized ACS patients as a secondary prevention. A supporting previous study found that in subgroups defined by age, gender, atrial fibrillation, dementia, diabetes, heart failure, revascularization, prior statin use, or use of other evidence-based drugs, a higher first statin dose after MI was associated with improved long-term outcomes [31]. A prior investigation identified the intensity of pre-hospital statins as the primary determinant correlated with the intensity of the post-discharge statin regimen [10]. An adjustment in the dosage of the statin therapy was similarly impacted by the pre-event statin dosage one year after discharge [10, 32].

Based on the one-way sensitivity analysis result, the long-term use of HIS among post-hospitalized ACS patients to prevent myocardial infarction and revascularization recurrence is preferable. Regardless of atherothrombotic risk classification, high-intensity statin medication at discharge after an acute myocardial infarction was linked with fewer major adverse cardiovascular events at five years, with the biggest absolute reduction reported in the high-risk TRS-2P class (Thrombolysis In Myocardial Infarction Risk Score for Secondary Prevention) [33]. This study result aligns with a few recent studies with retrospective database analyses, which showed the benefit of high-intensity statin use for secondary prevention of cardiac arrest, recurrent MI, and the need for revascularization [27, 34]. Thus, HIS is highly recommended for secondary prevention to be applied among post-hospitalized ACS patients in Indonesia.

Patients who are readmitted to the hospital with acute coronary syndrome (ACS) and fail to adhere to high-intensity statin therapy may face an increased likelihood of experiencing adverse cardiovascular events such as myocardial infarction, stroke, and cardiovascular mortality. Patients with ACS who fail to adhere to lifelong consuming HIS may incur higher healthcare expenses as a result of the necessity for more frequent and expensive interventions to manage cardiovascular events. In addition to the patients themselves, their families may also experience a decline in quality of life due to an increased likelihood of cardiovascular events and complications [35, 36].

Undertaking a cost-effectiveness analysis (CEA) on the lifetime utilization of high-intensity statins by patients who have been hospitalized with acute coronary syndrome (ACS) and are residing in low–middle-income countries (LMICs) can yield significant insights regarding the economic advantages associated with this therapeutic approach. A reduction in the likelihood of cardiovascular events such as all-cause mortality, myocardial infarction, stroke, rehospitalization, and revascularization is among the advantages of high-intensity statin therapy. Understanding the cost-effectiveness of high-intensity statin therapy can assist decision makers in LMICs, where healthcare resources are frequently scarce and cardiovascular disease prevalence is high, in the more efficient and effective allocation of resources. The incremental cost-effectiveness ratio (ICER) of high-intensity statin therapy compared to conventional-dose statin therapy can be calculated with the assistance of the CEA. This ICER can inform resource allocation and treatment strategy decisions. Additionally, by comparing the costs and outcomes of high-intensity statin therapy to the expenses associated with managing cardiovascular events without statin therapy, the CEA can assist in identifying potential cost-saving opportunities. For patients with ACS in LMICs, these data can be utilized to guide the development of cost-effective treatment strategies and inform health policy decision makers [26, 37, 38].

It is expected that the results of this research will give health policymakers an idea of the many benefits that can be obtained from both the financing aspect and the quality of life of patients with ACS. This study can help health policymakers make the right decisions regarding resource allocation for health service interventions, namely the lifelong use of HIS in ACS patients. The lifelong use of HIS as secondary prevention in ACS patients, which has been proven through this cost simulation model, can reduce costs caused by the occurrence of major adverse cardiac events after hospitalization, including death. If the financing for HIS therapy can be covered in its entirety by the Indonesian government's health insurance, then this could be a big step for the Indonesian government to reduce patient mortality due to ACS. This study is limited to secondary hospitals with 2nd Class facilities based on INA-CBGs guidelines. It is also supposed to apply to higher-class facilities and tertiary hospitals with higher budget coverage from Indonesia National Health Insurance. There have been very few previous cohort studies that have evaluated the financial benefits and quality of life in post-ACS patients who have been exposed to lifelong HIS use and its association with the risk of cardiac events. As a result, our work generates a CEA simulation using limited previous study data. Future cohort studies are needed on using HIS in LMICs, particularly in Indonesia, and financing lifelong HIS consumption through Indonesian national health insurance.

## Conclusion

A cost-effectiveness ratio (ICER) of Rp 31.843.492 per QALY gained indicates that the use of high-intensity statins among post-hospitalized patients with acute coronary syndrome in Indonesia is regarded to be effective. The use of high-intensity statins as a secondary prevention strategy among patients with ACS in order to prevent recurrences of myocardial infarction and revascularization is advised over a prolonged period of time.

## Recommendation

The findings of this trial strongly support the lifetime use of high-intensity statins (HISs) for post-hospitalized ACS patients in order to prevent future major adverse cardiac events. Given the higher cost and greater benefit of HIS over low–moderate-intensity statin (LMIS), the Indonesian government should include budget in the National Health Scheme to cover HIS treatment for ACS patients. Additionally, budget impact analysis is warranted to guide budget allocation for using HIS for post-hospitalized ACS patients. Furthermore, implementing a clinical pathway, improving healthcare insurance coverage, and improving medical literacy in both physicians and patients are all needed to support the use of statins at the recommended dosage among post-hospitalized patients with ACS in Indonesia. A future study in Indonesia should be conducted to assess the economic value of employing HIS for primary prevention among high-risk patients because it is not covered by current National Health Scheme.

## Data Availability

The data that support the findings of this study are available from the corresponding author upon reasonable request.

## Abbreviations

DALY: Disability-adjusted life year
IHD: Ischemic heart disease
ACS: Acute coronary syndromes
ASMRs: Age-standardized mortality rates
ASCVD: Atherosclerotic cardiovascular disease
CVD: Cardiovascular disease
LDL-C: Low-density lipoprotein-cholesterol
HIS: High-intensity statin
INA-CBGs: Indonesian Case Base Groups
LMIS: Low-to-moderate-intensity statin
QALY: Quality-adjusted life year
BPJS: Indonesian government insurance
RA: Resolved ACS
MI: Myocardial infarction
RV: Revascularization
CAr: Cardiac arrest
MACE: Major adverse cardiac event
QoL: Quality of life
ICER: Cost-effectiveness ratio
PSA: Probabilistic sensitivity analysis
WTP: Willingness-to-pay
WHO’s: World Health Organization’s
CHOICE: Choosing interventions that are cost-effective
GDP: Gross domestic product
CEA: Cost-effectiveness analysis
LMICs: Low–middle-income countries

## References

[1] Roser M, Ritchie H, Spooner F (2017) Burden of Disease. Available from: https://ourworldindata.org/burden-of-disease.

[2] Bueno H, Camm AJ, Lüscher TF, Maurer G, Serruys PW, James S (2018). Epidemiology of acute coronary syndromes. The ESC textbook of cardiovascular medicine.

[3] Arnett DK, Blumenthal RS, Albert MA, Buroker AB, Goldberger ZD, Hahn EJ (2019). ACC/AHA guideline on the primary prevention of cardiovascular disease: a report of the American College of Cardiology/American Heart Association Task Force on Clinical Practice Guidelines. Circulation.

[4] Timmis A, Kazakiewicz D, Townsend N, Huculeci R, Aboyans V, Vardas P (2023). Global epidemiology of acute coronary syndromes. Nat Rev Cardiol.

[5] Mach F, Baigent C, Catapano AL, Koskinas KC, Casula M, Badimon L (2019). ESC/EAS Guidelines for the management of dyslipidaemias: lipid modification to reduce cardiovascular risk. Eur Heart J Cardiovasc Pharmacother.

[6] Ruscica M, Macchi C, Pavanello C, Corsini A, Sahebkar A, Sirtori CR (2018). Appropriateness of statin prescription in the elderly. Eur J Intern Med.

[7] Sigglekow F, Horsburgh S, Parkin L (2020). Statin adherence is lower in primary than secondary prevention: a national follow-up study of new users. PLoS ONE.

[8] Grundy SM, Stone NJ, Blumenthal RS, Braun LT, Heidenreich PA, Lloyd-Jones D (2021). High-intensity statins benefit high-risk patients: why and how to do better. Mayo Clin Proc.

[9] Kim J, Park KT, Jang MJ, Park TK, Lee JM, Yang JH (2018). High-intensity versus non-high-intensity statins in patients achieving low-density lipoprotein cholesterol goal after percutaneous coronary intervention. J Am Heart Assoc.

[10] Rosenson RS, Kent ST, Brown TM, Farkouh ME, Levitan EB, Yun H (2015). Underutilization of high-intensity statin therapy after hospitalization for coronary heart disease. J Am Coll Cardiol.

[11] Lee SJ, Kang WC, Lee JY, Lee JB, Yang TH, Yoon J (2023). Treat-to-target versus high-intensity statin treatment in patients with or without diabetes mellitus: a pre-specified analysis from the LODESTAR trial. EClinicalMedicine.

[12] Indonesian Health Minister (2016) Peraturan Menteri Kesehatan No.64 Tentang Standar Tarif Pelayanan Kesehatan Dalam Penyelenggaraan Program Jaminan Kesehatan [Minister of Health Regulation No. 64 Concerning Standard Health Service Tariffs in the Implementation of Health Insurance Programs]. Jakarta: Indonesian Ministry of Health

[13] Kim DD, Silver MC, Kunst N, Cohen JT, Ollendorf DA, Neumann PJ (2020). Perspective and costing in cost-effectiveness analysis, 1974–2018. Pharmacoeconomics.

[14] Abbott JH, Wilson R, Pryymachenko Y et al (2022) Economic evaluation: a reader’s guide to studies of cost-effectiveness. Arch Physiother 12(28) 10.1186/s40945-022-00154-1 PMC9753355 36517825

[15] Neumann PJ (2009). Costing and perspective in published cost-effectiveness analysis. Med Care.

[16] Husereau D, Drummond M, Augustovski F, de Bekker-Grob E, Briggs AH, Carswell C (2022). Consolidated Health Economic Evaluation Reporting Standards 2022 (CHEERS 2022) statement: updated reporting guidance for health economic evaluations. BMC Med.

[17] Gray AM, Clarke PM, Wolstenholme JL, Wordsworth S (2011). Applied methods of cost-effectiveness analysis in healthcare.

[18] Gidwani R, Russell LB (2020). Estimating transition probabilities from published evidence: a tutorial for decision modelers. Pharmacoeconomics.

[19] Taylor DC, Pandya A, Thompson D, Chu P, Graff J, Shepherd J (2009). Cost-effectiveness of intensive atorvastatin therapy in secondary cardiovascular prevention in the United Kingdom, Spain, and Germany, based on the Treating to New Targets study. Eur J Health Econ.

[20] Lin FJ, Shyu KG, Hsieh IC, Huey-Herng Sheu W, Tu ST, Yeh SJ, Chen CI, Lu KC, Wu CC, Shau WY, Inocencio TJ, Wen YC, Yeh HI (2020). Cost-effectiveness of statin therapy for secondary prevention among patients with coronary artery disease and baseline LDL-C 70–100 mg/dL in Taiwan. J Formos Med Assoc.

[21] Barrios V, Kaskens L, Castellano JM, Cosin-Sales J, Ruiz JE, Zsolt I (2017). Usefulness of a cardiovascular polypill in the treatment of secondary prevention patients in Spain: a cost-effectiveness study. Rev Esp Cardiol.

[22] Almalki ZS, Guo JJ, Alahmari A, Alotaibi N, Thaibah H (2018). Cost-effectiveness of simvastatin plus ezetimibe for cardiovascular prevention in patients with a history of acute coronary syndrome: analysis of results of the IMPROVE-IT trial. Heart Lung Circ.

[23] Gómez-Gerique JA, Casciano R, Stern L, Rejas J (2004). A pharmacoeconomic evaluation of the of the effects of atorvastatin on early recurrent coronary syndromes in Spain. Eur J Health Econ.

[24] Ademi Z, Reid CM, Hollingsworth B, Stoelwinder J, Steg PG, Bhatt DL (2011). Cost-effectiveness of optimizing use of statins in Australia: using outpatient data from the REACH Registry. Clin Ther.

[25] Lazar LD, Pletcher MJ, Coxson PG, Bibbins-Domingo K, Goldman L (2011). Cost-effectiveness of statin therapy for primary prevention in a low-cost statin era. Circulation.

[26] Chan PS, Nallamothu BK, Gurm HS, Hayward RA, Vijan S (2007). Incremental benefit and cost-effectiveness of high-dose statin therapy in high-risk patients with coronary artery disease. Circulation.

[27] Rockberg J, Jørgensen L, Taylor B, Sobocki P, Johansson G (2017). Risk of mortality and recurrent cardiovascular events in patients with acute coronary syndromes on high intensity statin treatment. Prev Med Rep.

[28] Marseille E, Larson B, Kazi DS, Kahn JG, Rosen S (2015). Thresholds for the cost-effectiveness of interventions: alternative approaches. Bull World Health Organ.

[29] Kristina SA, Endarti D, Wiedyaningsih C, Fahamsya A, Faizah N (2018). Health care cost of noncommunicable diseases related to smoking in Indonesia, 2015. Asia Pac J Public Health.

[30] Andayani TM, Endarti D, Kristina SA, Rokhman MR (2017). Metode Untuk Memperkirakan Willingness-To-Pay per Quality Adjusted Life Year Sebagai Cost-Effectiveness Threshold [Methods for Estimating Willingness-To-Pay per Quality Adjusted Life Year as a Cost-Effectiveness Threshold]. Jur Manaj Pelay Farm.

[31] Kytö V, Rautava P, Tornio A (2023). Initial statin dose after myocardial infarction and long-term cardiovascular outcomes. Eur Heart J Cardiovasc Pharmacother.

[32] Xie G, Myint PK, Sun Y, Li X, Wu T, Gao RL (2022). Associated factors for discontinuation of statin use one year after discharge in patients with acute coronary syndrome in China. BMJ Open.

[33] Desjobert E, Tea V, Schiele F, Ferrières J, Simon T, Danchin N (2021). Clinical outcomes with high-intensity statins according to atherothrombotic risk stratification after acute myocardial infarction: the FAST-MI registries. Arch Cardiovasc Dis.

[34] Castillo-Costa YB, Mauro VM, Charask AA, Fairman E, Buhezo H, Barrero C (2018). Use of high-intensity statin strategy. Are the guidelines followed?. Revista Argentina de Cardiologia.

[35] Diaz R, Li QH, Bhatt DL et al (2020) Intensity of statin treatment after acute coronary syndrome, residual risk, and its modification by alirocumab: insights from the ODYSSEY OUTCOMES trial. Eur J Prev Cardiol 0(0) 10.1177/2047487320941987 33755145

[36] Sulzgruber P, Sinkovec H, Kazem N, Hofer F, Hammer A, Koller L, Todorovic M, Katsch F, Gall W, Duftschmid G, Heinze G, Niessner A (2020) Adherence to high-intensity statin therapy after acute coronary syndrome and its impact on patient outcome. Eur Heart J 41(2)

[37] Husain MJ, Spencer G, Nugent R, Kostova D, Richter P (2022). The cost-effectiveness of hyperlipidemia medication in low- and middle-income countries: a review. Glob Heart.

[38] Nherera L, Calvert NW, Demott K, Humphries SE, Neil HA, Minhas R, Thorogood M (2010). Cost-effectiveness analysis of the use of a high-intensity statin compared to a low-intensity statin in the management of patients with familial hypercholesterolaemia. Curr Med Res Opin.

